# Estimation of Drug Pharmacokinetics from Breast Feeding: A Simple Method Based on Meta-analysis

**DOI:** 10.9734/jamps/2019/v21i230126

**Published:** 2019-08-03

**Authors:** Oumar Aboubacar Alassane, De Pablos-Martinez Carlos, Maiga Mamoudou, Dao Sounkalo, Chatelut Etienne, Gandia Peggy

**Affiliations:** 1 Pharmacokinetics and Toxicology Laboratory, Federative Institute of Biology, Purpan University Hospital, Toulouse, France.; 2 Pharmacology and Pharmacogenetic Laboratory, University Institute of Cancer Oncopôle, Toulouse, France.; 3 HIV/TB Research and Training Center, University of Science, Techniques and Technologies in Bamako, Mali.; 4 Northwestern University, Division of Infectious Diseases, Chicago, USA.

**Keywords:** Breast milk, antiretroviral drugs, newborn exposure

## Abstract

**Background:**

In resource-limited settings, breastfeeding is the healthiest source of nutrition for newborns. For economic/cultural reasons, breastfeeding is the preferred option for the majority of mothers, including HIV-positive mothers.

**Objective:**

The objective of this review is to document parameters characterizing antiretroviral therapy (ARV) diffusion into breast milk associated with the estimated ARV amount ingested by breastfed infant and clinical/biological abnormalities.

**Data Source and Eligibility Criteria:**

Twenty seven (27) published articles on the aspects of Pharmacokinetic parameters on ARV diffusion into breast milk have shown a large variability without clear interpretation on drugs diffusion. Using PubMed and Embase, we conducted a search to identify all published studies at 2015 that characterized antiretroviral drug diffusion from mother to infant via breast milk. We identified 27 published studies that characterized antiretroviral drug passage from mother to infant (drug concentrations in mother’s milk and breastfed plasma). Information was sufficiently complete for inclusion in the present analysis for only six antiretroviral drugs.

**Results:**

Finally, only data for nevirapine and efavirenz were exploitable because some of the studies found null or non-detectable levels, which were not suitable for simulations. Median (IQR) nevirapine CL/F were 0.022 (0.013–0.038) for newborns, 0.121 (0.116–0.125) for children and 0.056 (0.045–0.070) for mothers, all in L/h/kg. Efavirenz CL/F were 0.025 (0.016–0.039) for newborns, 0.273 (0.261–0.285) for children and 0.160 (0.153–0.167) for mothers, also in L/h/kg.

**Conclusion:**

Pharmacokinetics parameters of efavirenz and nevirapine are important to be determined in breastfed newborns.

## 1. INTRODUCTION

In newborns, exploring drug pharmacokinetic is exceptionally performed because newborns represent a physiologically fragile population in which drug administration has to be limited. However, in some conditions, newborns can be indirectly exposed to drugs and consequently to the associated risk of adverse events, as is the case during breastfeeding. In this situation, the current practice consists in monitoring adverse effects in the newborn and in determining drug concentrations in the milk and sometimes in the newborn’s plasma to have information on the newborn’s drug exposure. Such information helps pediatricians to decide to either maintain or stop breastfeeding when mothers are treated [1,2]. In addition, milk and newborn’s plasma concentrations can be used to estimate some pharmacokinetic (PK) parameters of the ingested drug, including the apparent clearance (Cl/F). Apparent clearance is a hybrid pharmacokinetic parameter combining information on both absorption and elimination. Unfortunately, Cl/F is not often estimated and compared with values reported in infants and adults. Such comparison would bring information on the PK behavior of the drug at the beginning of the patient’s life. PK behavior is essential information for some drugs, especially those with a low therapeutic index, large inter-individual PK, low intra-individual PK variability and a pharmacokinetic-pharmacodynamics relationship. For these kind of drugs, the administered dose is determined taking into account the target exposure and the PK parameters of each patient.

Paired breast milk and newborn’s plasma samples are necessary to estimate Cl/F and are mainly available for antiretroviral drugs in developing countries. Indeed, the benefits of breastfeeding [3–5] are particularly notable in developing countries, where no or suboptimal breastfeeding was the attributable cause of over 800,000 deaths, 11.6% of all deaths, among children under five years of age in 2011 [6]. The increased risk of morbidity and mortality from diarrheal illness and pneumonia among non-breastfed infants has clearly been established [7, 8]. The World Health Organization (WHO) and the Funds of the United Nations for children (UNICEF) recommend exclusive breastfeeding for the first six months of life, then the introduction of supplementary foods and continuous breastfeeding up to at least two years of age [9]. This recommendation for non HIV-infected mothers does apply to HIV-infected mothers, except continuous breastfeeding lasts only up to age 12 months or as soon as the child can be assured a nutritionally adequate and safe diet without breast milk [10]. To prevent mother-to-child HIV transmission during breastfeeding, the WHO recommends maternal use of triple antiretroviral therapy (ART) [11]. Although it is a highly effective strategy to reduce mother-to-child HIV transmission, it does increase an infant’s exposure to antiretroviral drugs during breastfeeding. ARV diffusion into the breast milk has subsequent risk of adverse events for infants [12–15]. Therefore, the WHO has identified surveillance antiretroviral-related toxicities in infants during the breastfeeding, as well as effects of ARV exposure on a child’s growth and development, as an area of critically needed focus [16].

In breastfed newborns, in addition to the clinical survey, the exploration of the pharmacokinetic behavior of ARV is essential because this special population cannot be considered “small adults” from physiological and pharmacokinetic points of view. The primary aim of this paper was to estimate in newborns the apparent clearance of two commonly prescribed antiretrovirals (efavirenz and nevirapine) to HIV-infected breastfeeding mothers in developing countries. Secondly, the CL/F of these two drugs was compared with the values reported in children and adults.

## 2. MATERIALS AND METHODS

**Fig. 1.**
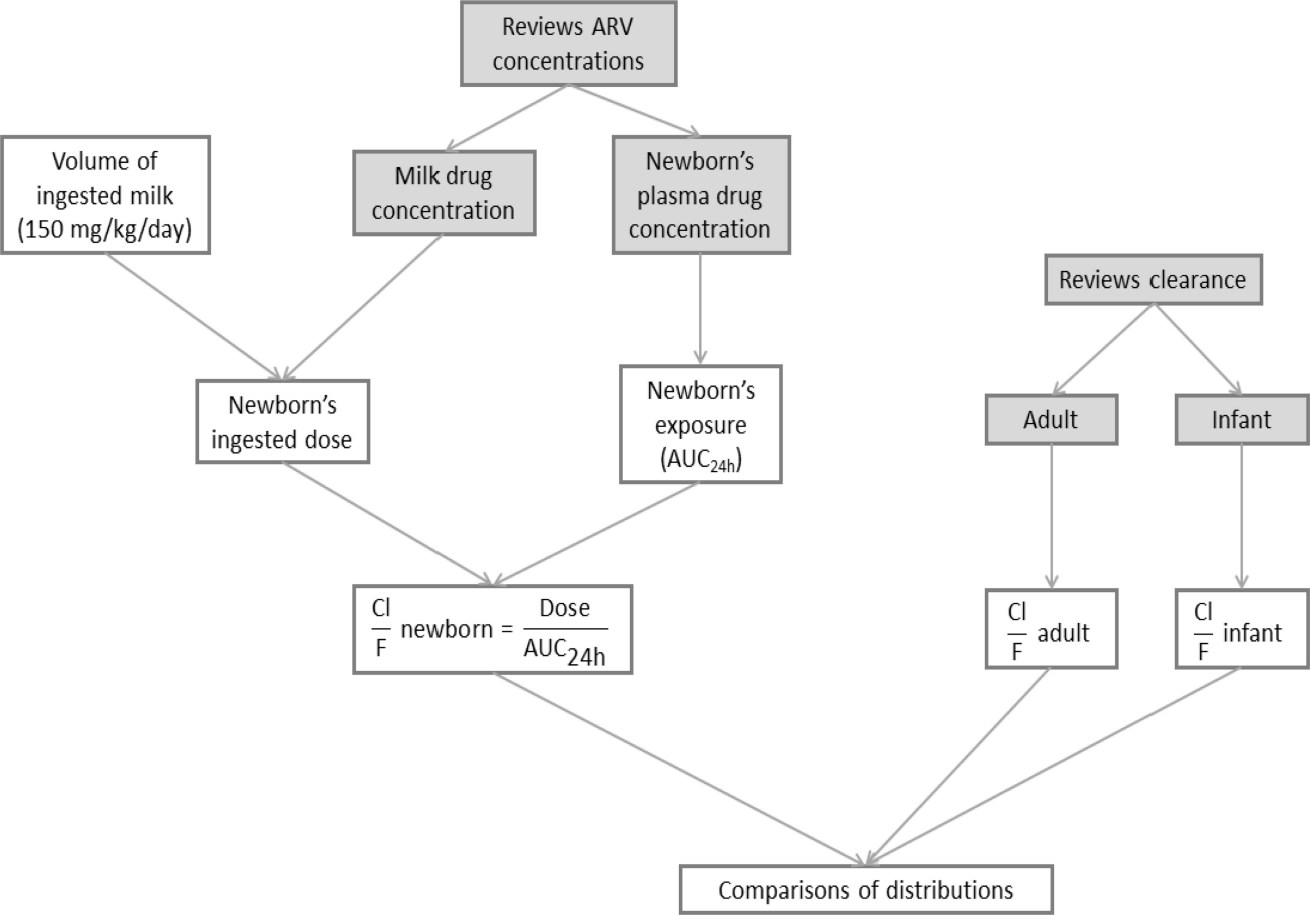
Estimation of newborn’s and mother’s apparent clearance

Using PubMed and Embase, we conducted a search to identify all published studies at 2015 that characterized antiretroviral drug diffusion from mother to infant via breast milk. To comply with study inclusion criteria, published findings had to provide information on antiretroviral drug concentration in paired breast milk and infant plasma, with values described as either mean and standard deviation (SD) or median and interquartile range (IQR) (Fig. 1).

Monte Carlo simulations (20 000) were performed for milk and newborn’s plasma concentrations using the Excel program [17,18], applying a normal distribution. Volumes of breast milk ingested by the breastfed infant were also simulated (20 000 simulations), using an average volume of 150 ± 20 mL/kg/day (Fig. 1).

The newborn ingested dose (NID) was calculated applying the following formula: *NID = C*_*milk*_ × *V*_*i*_, where C_milk_ is the drug milk concentration and V_i_ is the milk volume ingested by the newborn. Newborn exposure (AUC_24h_) was calculated by multiplying the newborn’s plasma drug concentration for 24 hours (*AUC*_24*h*_ = *C*_*P*_ × 24*h*) (Fig. 1).

The CL/F (L/kg) was calculated by the following formula: $CL/F=\frac{NID}{AUC_{24h}}.$ For adults and children, 20 000 Monte Carlo simulations were applied to CL/F values from the literature [19–23] (Fig. 1).

RStudio freeware (version 0.99.489, RStudio Inc.) was used to compare the density distributions of the simulations.

## 3. RESULTS

**Fig. 2.**
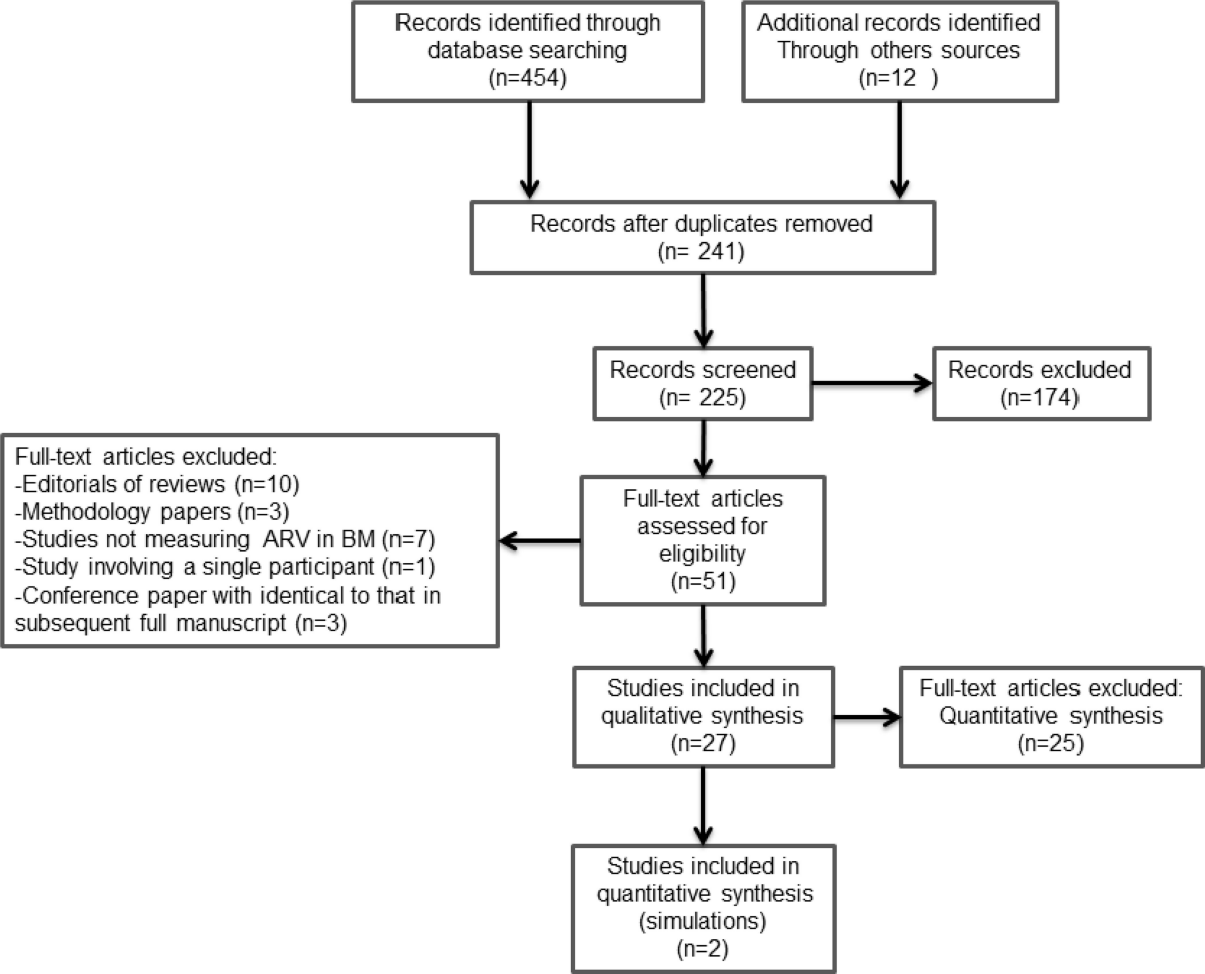
Flow diagram of article selection during review process

We identified 27 published studies that characterized antiretroviral drug passage from mother to infant (drug concentrations in mother’s milk and breastfed plasma). Information was sufficiently complete for inclusion in the present analysis for only six antiretroviral drugs (flow diagram of studies selected is represented in Fig. 2).

Selected molecules concerned two nucleoside reverse transcriptase inhibitors (NRTIs), stavudine and lamivudine; two non-nucleoside reverse transcriptase inhibitors (NNRTIs), nevirapine and efavirenz; and one pharmacokinetically-enhanced protease inhibitor, lopinavir/ritonavir. Finally, only data for nevirapine and efavirenz were exploitable because some of the studies [13,24] found null or non-detectable levels which were not suitable for simulations. The data obtained from bibliography and used for simulations are collected in Table 1.

**Fig. 3.**
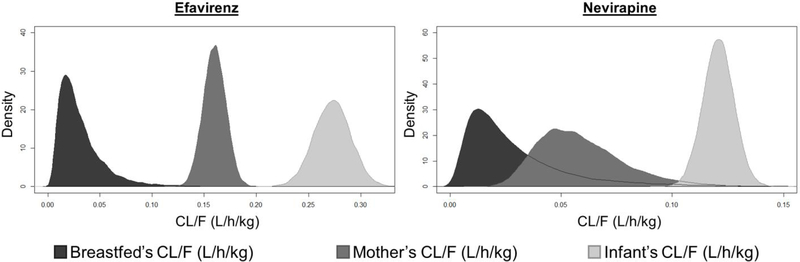
Distribution of simulated breastfed newborns’, mothers’ and infants’ apparent clearances

Median (IQR) nevirapine CL/F were 0.022 (0.013–0.038) for newborns, 0.121 (0.116–0.125) for children and 0.056 (0.045–0.070) for mothers, all in L/h/kg. Efavirenz CL/F were 0.025 (0.016–0.039) for newborns, 0.273 (0.261–0.285) for children and 0.160 (0.153–0.167) for mothers, also in L/h/kg. The density distribution of the simulations results are shown in Fig. 3.

## 4. DISCUSSION

In this study, we tried to present a simple approach to estimate newborn’s apparent clearance from paired mother’s milk and breastfed newborn’s plasma samples. Apparent clearances *per* kilogram were largely superior for infants than for breastfed newborns (5-fold greater for nevirapine and 10-fold greater for efavirenz). Mothers’ apparent clearances were also superior to those of breastfed newborns for nevirapine (2.5-fold) and efavirenz (more than 5fold) [25].

In the case of efavirenz, we used children’ and adults’ clearances estimated by the model published by Salem et al. [19]. These authors validated a base model using children’ samples, for children aged from 2 months to 16 years old. Later, this base model was allometrically scaled to a weight of 70 kg leading to a final model used for the prediction of efavirenz pharmacokinetics in adults. In the same study, the authors established that 90% of efavirenz metabolism maturity was not reached until 9 months. This is coherent with the low apparent clearances that we estimated for breastfed newborns. Moreover, higher CL/F *per* kg in children (0.19 L/h/kg; 0.21–0.26 L/h/kg) than in adults (0.15 L/h/kg) were also reported by other authors [26, 27]. These results seem to be explained by the fact that efavirenz is mainly metabolized by the enzyme CYP450 2B6, the expression of which increases with age [28]. However, CYP2B6 expression does not completely explain the fact that adults present lower apparent clearances compared with children. Consequently, modifications of efavirenz diffusion through the intestinal wall between childhood and adulthood cannot be excluded.

With nevirapine, Nikanjam et al. [29] reported that apparent clearance was lower during the first year of life while the values increase after the first year and remain stable from 1 to more than 12 years old. In a previous review published by Hoody and Fletcher [27], CL/F was found to be the same for adults and children older than 8 years (0.060 L/h/kg), but it was 2-fold higher for infants from 9 months to 8 years (0.120 L/h/kg). For newborns from 48 to 72 hours of life, CL/F was 0.0361 L/h/kg. All these values are consistent with our results. Nevirapine is mainly metabolized by CYP3A5 in African populations [30,31]. However, as de Wildt et al. [32] did not show any change in CYP3A5 enzymatic activity related to age, the mechanisms implicated in the age-based changes in apparent clearance are still unclear.

While our results underline the difference between newborns’ and children’ apparent clearance for antiretrovirals, we are aware of the limitations of our study. Firstly, quantifying the volume of ingested milk is challenging. Thus, we used the standard assumption of 150 mL/kg/day breast milk intake. Because we do not have any information on the actual distribution of consumption, we proposed a range of 20 mL/kg/day among and below 150 mL/kg/day for this distribution [2]. Secondly, the mothers’ and children’ apparent clearances, used to compare with newborns’ apparent clearances, were obtained from different populations. For efavirenz, the selected population (adults and children) came from the United States with a 50% Afro-American population. For nevirapine, the adult population was Dutch while the children were from Kenya. We cannot exclude a possible inter-population pharmacokinetic variability. To improve our approach, it will be necessary to use children and adult data from the same population as that of newborns. Finally, breastfed newborn’s ingested dose and plasma AUC were estimated from a single time point during the dosing interval. Our computation assumes a constant concentration of drug in ingested breast milk as well as in breastfed infant plasma throughout the dosing interval. This assumption seems reasonable for two reasons: (i) drug concentration in both matrices (breast milk and breastfed infant plasma) is supposed to be at steady state due to the repeated drug ingestion by the mother; [33] low variation of drug concentration is expected in ingested milk due to the short duration of breastfeeding (+/− 20 minutes of suckling) as well as in breastfed infant plasma due to repeated sucklings throughout the dosing interval (6–8 sucklings/day) mimicking a perfusion.

## 5. CONCLUSION

Our approach enabled us to estimate the apparent clearance in newborns and to obtain some information on the pharmacokinetic behavior of drugs in this special population who cannot be considered as “small adults”.

## Figures and Tables

**Table 1.** Data used for simulations

		Efavirenz	Nevirapine

New born	C_milk_ (mg/mL)	3.51±1.72^1^	2.901 (2.097–4.684)^2^
	C_p_ breastfed (mg/mL)	0.86±0.41^1^	0.809 (0.535–1.061)^2^
	Reference	[25]	[13]

Infant	CL/F (L/h)	5.1 (6.1 %)^3^	1.81 (5.7%)^3^
	Weight (kg)	18,7 *	15 *
	Reference	[19]	[23]

Adult	CL/F (L/h)	11.2 (6.8%)^3^	3.93 (2.76–4.32)^2^
	Weight (kg)	70 **	70 **
	Reference	[19]	[20]

1 Average±SD;

2 Median (IQR);

3 Average (RSE%);

* Median value;

** Standard weight

## References

[1] Breastfeeding and the use of human milk. American Academy of Pediatrics. Work Group on Breastfeeding. Pediatrics. 1997; 100(6):1035–9. 9411381 10.1542/peds.100.6.1035

[2] Eidelman AI. Breastfeeding and the use of human milk: An analysis of the American Academy of Pediatrics 2012 Breastfeeding Policy Statement. Breastfeed Med. 2012; 7(5):323–4. 22946888 10.1089/bfm.2012.0067

[3] Ip S, Chung M, Raman G, Trikalinos TA, Lau J. A summary of the agency for healthcare research and quality’s evidence report on breastfeeding in developed countries. Breastfeed Med. 2009;4(Suppl 1):S17–30. 19827919 10.1089/bfm.2009.0050

[4] Horta BL, Victoria CG. Long-term effects of breastfeeding: A systematic review In: Organization WH, ed. GENEVA: WHO; 2013.

[5] Eidelman AI, Schanler RJ. American academy of pediatrics section on breastfeeding. Breastfeeding and the Use of Human Milk. Pediatrics. 2012;129(3): e827–e41. 22371471 10.1542/peds.2011-3552

[6] Black RE, Victora CG, Walker SP, Bhutta ZA, Christian P, de Onis M, Maternal and child undernutrition and overweight in low-income and middle-income countries. Lancet. 2013;382(9890):427–51. 23746772 10.1016/S0140-6736(13)60937-X

[7] Lamberti LM, Fischer Walker CL, Noiman A, Victora C, Black RE. Breastfeeding and the risk for diarrhea morbidity and mortality. BMC Public Health. 2011; 11(Suppl 3):S15. 10.1186/1471-2458-11-S3-S15 PMC3231888 21501432

[8] Lamberti LM, Zakarija-Grkovic I, Fischer Walker CL, Theodoratou E, Nair H, Campbell H, Breastfeeding for reducing the risk of pneumonia morbidity and mortality in children under two: a systematic literature review and metaanalysis. BMC Public Health. 2013;13 (Suppl 3):S18. 24564728 10.1186/1471-2458-13-S3-S18 PMC3847465

[9] WhO/uNiCEF. Global strategy on infant and young child feeding. In: WHO, ed. GENEVA: WHO; 2003.

[10] WHO/, UNAIDS/, UNFPA/, UNICEF. Guidelines on HIV and infant feeding Principles and recommendations for infant feeding in the context of HIV and a summary of evidence. . GENEVA: WHO; 2010. 24501786

[11] WHO. Consolidated guidelines on the use of antiretroviral drugs for treating and preventing HIV infection Recommendations for a public health approach. GENEVA: WHO 2013;272. 24716260

[12] Zeh C, Weidle PJ, Nafisa L, Lwamba HM, Okonji J, Anyango E, HIV-1 drug resistance emergence among breastfeeding infants born to HIV-infected mothers during a single-arm trial of triple-antiretroviral prophylaxis for prevention of mother-to-child transmission: a secondary analysis. PLoS Med. 2011;8(3):e1000430. 10.1371/journal.pmed.1000430 PMC3066134 21468304

[13] Palombi L, Pirillo MF, Andreotti M, Liotta G, Erba F, Sagno JB, Antiretroviral prophylaxis for breastfeeding transmission in Malawi: Drug concentrations, virological efficacy and safety. Antiviral Therapy. 2012;17(8):1511–9. 22910456 10.3851/IMP2315

[14] Fogel J, Li Q, Taha TE, Hoover DR, Kumwenda NI, Mofenson LM, Initiation of antiretroviral treatment in women after delivery can induce multiclass drug resistance in breastfeeding HIV- infected infants. Clin Infect Dis. 2011;52(8): 1069–76. 21460326 10.1093/cid/cir008 PMC3070029

[15] Dryden-Peterson S, Shapiro RL, Hughes mD, Powis K, Ogwu A, Moffat C, Increased risk of severe infant anemia after exposure to maternal HAART, Botswana. J Acquir Immune Defic Syndr. 2011;56(5):428–36. 21266910 10.1097/QAI.0b013e31820bd2b6 PMC3112252

[16] WHO. March 2014 Supplement to the 2013 consolidated guidelines on the use of antiretroviral drugs for treating and preventing HIV infection Recommendations for a public health approach. . GENEVA: WHO; 2014. 24716260

[17] Bonate PL. A brief introduction to monte carlo simulation. Clin Pharmacokinet. 2001;40(1):15–22. 11236807 10.2165/00003088-200140010-00002

[18] Raychaudhuri S. Introduction to Monte Carlos Simulation Winter Simulation Conference. Austin, TX: IEEE 2008:91–100.

[19] Salem AH, Fletcher CV, Brundage RC. Pharmacometric characterization of efavirenz developmental pharmacokinetics and pharmacogenetics in HIV-infected children. Antimicrobial Agents and Chemotherapy. 2014;58(1):136–43. 24145522 10.1128/AAC.01738-13 PMC3910794

[20] Cooper CL, van Heeswijk RP. Once-daily nevirapine dosing: A pharmacokinetics, efficacy and safety review. HIV Medicine. 2007;8(1):1–7. 10.1111/j.1468-1293.2007.00426.x 17305925

[21] Benaboud S, Treluyer JM, Urien S, Blanche S, Bouazza N, Chappuy H, Pregnancy-related effects on lamivudine pharmacokinetics in a population study with 228 women. Antimicrobial Agents and Chemotherapy. 2012;56(2):776–82. 22106227 10.1128/AAC.00370-11 PMC3264256

[22] Piana C, Zhao W, Adkison K, Burger D, Jacqz-Aigrain E, Danhof M, Covariate effects and population pharmacokinetics of lamivudine in HIV- infected children. British Journal of Clinical Pharmacology. 2014;77(5):861–72. 24118070 10.1111/bcp.12247 PMC4004406

[23] Vreeman RC, Nyandiko WM, Liechty EA, Busakhala N, Bartelink IH, Savic RM, Impact of adherence and anthropometric characteristics on nevirapine pharmacokinetics and exposure among HIV- infected Kenyan children. J Acquir Immune Defic Syndr. 2014;67(3):277–86. 25140906 10.1097/QAI.0000000000000300

[24] Fogel JM, Taha TE, Sun J, Hoover DR, Parsons TL, Kumwenda JJ, Stavudine concentrations in women receiving postpartum antiretroviral treatment and their breastfeeding infants. J Acquir Immune Defic Syndr. 2012;60(5): 462–5. 22614899 10.1097/QAI.0b013e31825ddcfa PMC3404155

[25] Schneider S, Peltier A, Gras A, Arendt V, Karasi-Omes C, Mujawamariwa A, Efavirenz in human breast milk, mothers’, and newborns’ plasma. J Acquir Immune Defic Syndr. 2008;48(4):450–4. 18614925 10.1097/QAI.0b013e31817bbc21

[26] Fletcher CV, Brundage RC, Fenton T, Alvero CG, Powell C, Mofenson LM, Pharmacokinetics and pharmacodynamics of efavirenz and nelfinavir in HIV-infected children participating in an area-under-the-curve controlled trial. Clin Pharmacol Ther. 2008;83(2):300–6. 17609682 10.1038/sj.clpt.6100282 PMC2848440

[27] Hoody DW, Fletcher CV. Pharmacology considerations for antiretroviral therapy in human immunodeficiency virus (HIV)-infected children. Semin Pediatr Infect Dis. 2003;14(4):286–94. 14724793 10.1053/j.spid.2003.09.004

[28] Pearce RE, Gaedigk R, Twist GP, Dai H, Riffel AK, Leeder JS, Developmental Expression of CYP2B6: A comprehensive analysis of mrna expression, protein content and bupropion hydroxylase activity and the impact of genetic variation. Drug Metab Dispos; 2015. 10.1124/dmd.115.067546 PMC4931886 26608082

[29] Nikanjam M, Kabamba D, Cressey TR, Burger D, Aweeka FT, Acosta EP, Nevirapine exposure with WHO pediatric weight band dosing: Enhanced therapeutic concentrations predicted based on extensive international pharmacokinetic experience. Antimicrobial Agents and Chemotherapy. 2012;56(10):5374–80. 22869579 10.1128/AAC.00842-12 PMC3457371

[30] Dickinson L, Chaponda M, Carr DF, van Oosterhout JJ, Kumwenda J, Lalloo DG, Population pharmacokinetic and pharmacogenetic analysis of nevirapine in hypersensitive and tolerant HIV-infected patients from Malawi. Antimicrobial agents and chemotherapy. 2014;58(2):706–12. 24217698 10.1128/AAC.02069-13 PMC3910846

[31] Brown KC, Hosseinipour MC, Hoskins JM, Thirumaran RK, Tien HC, Weigel R, Exploration of CYP450 and drug transporter genotypes and correlations with nevirapine exposure in Malawians. Pharmacogenomics. 2012;13(1):113–21. 22111602 10.2217/pgs.11.132 PMC3292264

[32] de Wildt SN. Profound changes in drug metabolism enzymes and possible effects on drug therapy in neonates and children. Expert Opinion on Drug Metabolism & Toxicology. 2011;7(8):935–48. 21548840 10.1517/17425255.2011.577739

[33] Musoke P, Guay LA, Bagenda D, Mirochnick M, Nakabiito C, Fleming T, A phase I/II study of the safety and pharmacokinetics of nevirapine in HIV-1- infected pregnant Ugandan women and their neonates (HIVNET 006). AIDS. 1999; 13(4):479–86 10197376 10.1097/00002030-199903110-00006

